# Exogen-allergische Alveolitis: Die aktuelle S2k-Leitlinie aus radiologischer Sicht

**DOI:** 10.1007/s00117-025-01461-x

**Published:** 2025-06-17

**Authors:** Okka W. Hamer, Dirk Koschel, Beate Rehbock

**Affiliations:** 1 Institut für Röntgendiagnostik, Universitätsklinikum Regensburg und Lungenfachklinik Caritas St. Maria, Donaustauf, Deutschland; 2 Abteilung Innere Medizin und Pneumologie, Fachkrankenhaus Coswig, Lungenzentrum, Coswig und Bereich Pneumologie, Medizinische Klinik 1, Universitätsklinikum Carl Gustav Carus, TU Dresden, Dresden, Deutschland; 3 Privatpraxis und Radiologische Begutachtung mit Spezialgebiet Lunge, Potsdam, Deutschland

**Keywords:** Interstitielle Lungenerkrankung, High-Resolution-Computertomographie, Fibrose, Inflammation, Air-Trapping, Intertitial lung disease, High-resolution computed tomography, Fibrosis, Inflammation, Air trapping

## Abstract

Die exogen-allergische Alveolitis (EAA) ist eine seltene, auf die Lunge beschränkte Erkrankung, der eine immunologisch bedingte Entzündungsreaktion des Lungenparenchyms und der terminalen Bronchioli zugrunde liegt. Die erste deutsche Leitlinie zur Diagnostik und Therapie der EAA ist im Jahr 2024 erschienen. Die Bildgebungsmethode der Wahl ist die High-Resolution-Computertomographie (HRCT). Radiologisch wird eine rein inflammatorische von einer fibrotischen EAA unterschieden. Bei Anzeichen des fibrotischen Typs in der HRCT sollte das quantitative Verhältnis zwischen der Inflammation und der Fibrose bestimmt und im Befund angegeben werden. Die Prognose hängt stark vom Ausmaß der Fibrose und dem Anteil sog. Honigwaben ab.

## Abstract (en)

Hypersensitivity pneumonitis (HP, also known as extrinsic allergic alveolitis [EAA]) is a rare disease which is limited to the lungs and caused by an immunological inflammatory reaction of the lung parenchyma and terminal bronchioli. The first German guideline on the diagnosis and treatment of HP was published in 2024. The imaging method of choice is high-resolution computed tomography (HRCT). Radiologically, a distinction is made between purely inflammatory and fibrotic HP. If there are signs of fibrotic HP in the HRCT, the relation between the extent of inflammation and fibrosis should be determined and given in the report. The prognosis depends significantly on the extent of fibrosis and the proportion of so-called honeycombs.

Die exogen-allergische Alveolitis (EAA) ist eine auf die Lunge beschränkte immunologisch komplex (Typ III und IV) bedingte Entzündungsreaktion des Lungenparenchyms und der terminalen Bronchioli. Sie wird in der überwiegenden Mehrheit durch die Inhalation von Antigenen bei zuvor sensibilisierten und genetisch prädisponierten Personen hervorgerufen. Es gibt unzählige Antigene, die als Auslöser fungieren können [3]. Als klassische Krankheitsbilder seien die Vogelhalterlunge (Auslöser: Proteine in Federn, Serum und Kot von Vögeln), die Farmerlunge (Auslöser: Bakterien, z. B. *Thermoactinomyces vulgaris*, und Pilze, z. B. Aspergillus fumigatus in Heu, Stroh, verschimmelten Pflanzen) und die Befeuchterlunge (Auslöser: z. B. *Achromobacter* in kontaminierten Befeuchtern und Klimaanlagen) genannt. Das Muster der EAA kann jedoch auch nichtinhalativ im Rahmen eines medikamentös-toxischen Geschehens vorkommen (dann als Hypersensitivitätspneumonitis bezeichnet).

Die EAA weist unterschiedliche Verlaufsformen auf. Einerseits wurde nach der klinischen Symptomatik ein akuter von einem chronischen Verlauf unterschieden, andererseits in jüngeren Publikationen nur eine nicht-fibrotische von einer fibrotischen Verlaufsform [5, 10, 14, 17, 20].

Die aktuelle internationale Leitlinie unterteilt die EAA klinisch in einen nicht-fibrotischen und fibrotischen Typ [14]. Radiologisch wird hier für den *nicht-fibrotischen* Typ die Einteilung in High-Resolution-Computertomographie(HRCT)-Muster „typisch für EAA“ vs. „vereinbar mit EAA“ und für den *fibrotischen* Typ die Einteilung in HRCT-Muster „typisch für EAA“ vs. „vereinbar mit EAA“ vs. „unbestimmt“ vorgeschlagen.

Im Jahr 2024 ist die erste deutsche S2k-Leitlinie zur Diagnostik und Therapie der EAA veröffentlicht worden, die in wichtigen Punkten von der internationalen Leitlinie abweicht [9]. Im folgenden Übersichtsartikel werden die Inhalte der deutschen Leitlinie mit Fokus auf den radiologischen Part vorgestellt.

## Epidemiologie

Die EAA ist eine seltene Erkrankung mit einer Inzidenz von ca. 1 Neuerkrankung pro 100.000/Jahr [6, 15, 18]. Sie tritt in der überwiegenden Mehrheit bei Erwachsenen auf. Das mittlere Erkrankungsalter liegt bei 52 Jahren. Selten können auch Kinder betroffen sein [8, 19]. Im Gegensatz zu vielen anderen interstitiellen Lungenerkrankungen ist das Rauchen ein protektiver Faktor [18, 21]. Dies wird auf die immunsuppressiven Eigenschaften des Rauchens, vor allem des inhalierten Nikotins zurückgeführt.

## Klinische Einteilung

**Abb. 1:**
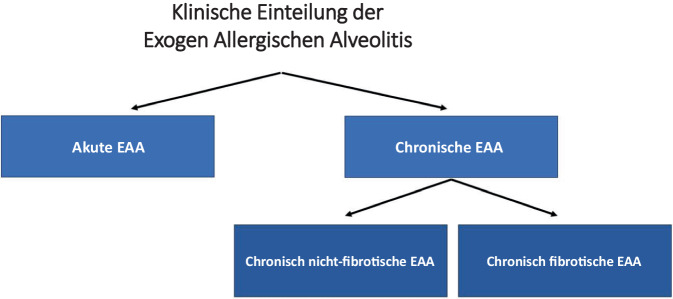
Klinische Einteilung der exogen-allergischen Alveolitis (EAA) gemäß S2k-Leitlinie

Klinisch macht es nach Ansicht der deutschen Leitlinienkommission weiterhin Sinn, die EAA in eine akute und chronische Verlaufsform einzuteilen. Bei der akuten Form kommt es 3–12 h nach wiederholter und intensiver Antigenexposition zu plötzlichen grippeähnlichen Symptomen. Bei Antigenkarenz ist der Patient in der Regel nach 24–48 h auch ohne medikamentöse Therapie wieder beschwerdefrei. Die chronische Form wird entweder durch eine lang andauernde kontinuierliche Antigenexposition oder durch rezidivierende Expositionen ausgelöst. Während die akute Form immer nicht-fibrotisch verläuft, werden bei der chronischen Form weitergehend die Subtypen fibrotisch und nicht-fibrotisch unterschieden (Abb. 1).

## Radiologische Einteilung

**Abb. 2:**
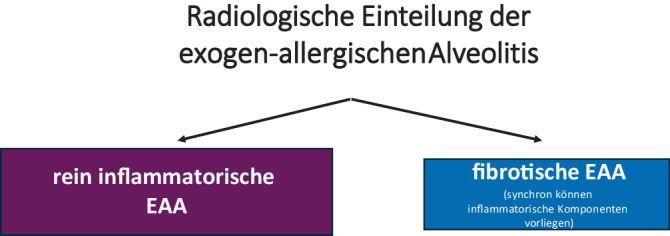
Radiologische Einteilung der exogen-allergischen Alveolitis (EAA) gemäß S2k-Leitlinie

Was die Kategorisierung der EAA in der Radiologie angeht, hat sich die internationale Leitlinie in Deutschland aufgrund ihrer Komplexität nicht durchgesetzt. In Anlehnung an die klinische Einteilung der deutschen S2k-Leitlinie wird aus radiologischer Sicht vorgeschlagen, in der HRCT pragmatisch zwischen einer rein inflammatorischen und einer fibrotischen Form zu unterscheiden, da diese Einteilung objektivierbar und sowohl therapeutisch als auch prognostisch relevant ist (Abb. 2). Dabei kann die fibrotische Form HRCT-morphologisch rein fibrotisch sein oder in Kombination mit inflammatorischen Zeichen auftreten. Umgekehrt kann die inflammatorische Form sowohl bei der akuten als auch bei der chronischen EAA gesehen werden, die fibrotische Form wird nur bei der chronischen EAA gesehen.

## Technik

Die bildgebende Modalität der Wahl ist die native dünnschichtige Volumen-Computertomographie (CT), auch als High-Resolution-CT (HRCT) bezeichnet. Auf die technischen Parameter soll nur kurz eingegangen werden:

1. Die ausgespielte Schichtdicke sollte 1,2 mm nicht überschreiten.
2. Neben der axialen Orientierung sind zusätzlich max. 1,2 mm dicke multiplanare Rekonstruktionen in sagittaler und koronarer Ebene empfohlen.
3. Zusätzliche Aufnahmen in Exspiration (zur besseren Erkennbarkeit des Air-Trappings, siehe unten) oder Bauchlage (zur Unterscheidung einer funktionellen Hypostase von einer realen Parenchymveränderung) können bei Bedarf entweder als Einzelscans oder in einer erneuten Spiralakquisition in Low-dose-Technik erfolgen.

## Morphologie in der High-Resolution-Computertomographie

**Abb. 3:**
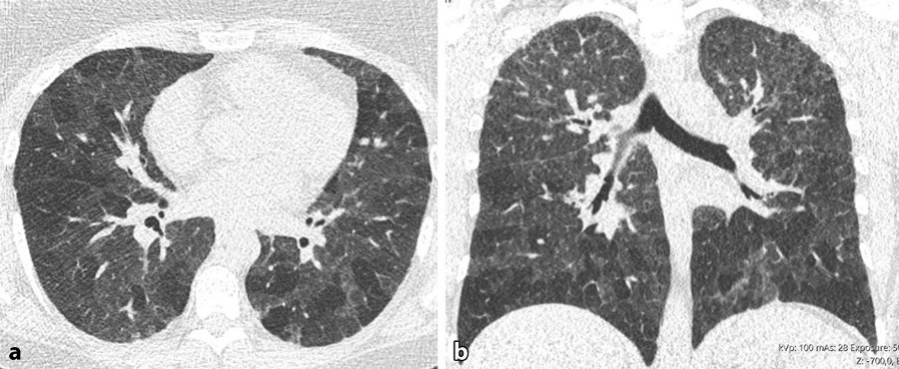
Inflammatorische exogen-allergischen Alveolitis (EAA). **a** Axiale und **b** koronare multiplanare Reformation (MPR) einer nativen High-Resolution-Computertomographie (HRCT). Diffus in der Lunge verteilt zeigt sich eine Kombination aus Milchglas und Air-Trapping. Zeichen einer Fibrose fehlen

Die EAA äußert sich in der HRCT in Form einer charakteristischen Kombination von HRCT-Zeichen und ihrer Verteilung in der Lunge (Tab. 1, 2 und 3 und Abb. 3, 4 und 5). Zu beachten ist, dass nicht alle aufgeführten Merkmale synchron vorliegen müssen. Meist liegt eine Auswahl in unterschiedlicher Kombination und Gewichtung vor. Grundsätzlich steigt die Wahrscheinlichkeit für das Vorliegen einer EAA an, je mehr Zeichen in typischer Verteilung vorliegen. Für die fibrotische EAA gilt, dass meist synchron Zeichen des inflammatorischen Stadiums vorliegen. Folgende Punkte sind ergänzend zu beachten:

1. Air-Trapping stellt sich in Form eines Mosaikmuster dar. Da ein Mosaikmuster verschiedene Ursachen haben kann, darf Air-Trapping streng genommen nur dann befundet werden, wenn exspiratorische Aufnahmen vorliegen [2, 4, 7]. Es äußert sich hier in Form von geographisch konfigurierten hypodensen Arealen, die im Vergleich zur inspiratorischen Aufnahme nicht adäquat an Dichte zunehmen und nicht adäquat an Volumen abnehmen. Häufig ist der Befund jedoch schon in Inspiration so suggestiv, dass auf eine exspiratorische Aufnahme verzichtet werden kann. Minimum-Intensitäts-Projektionen (MinIPs) erleichtern die Erkennbarkeit des Air-Trappings.
2. Das Drei-Dichte-Zeichen bezeichnet das Nebeneinander von 3 Graustufen im Lungenparenchym (Air-Trapping, normale Lunge, Milchglas/Konsolidierung; [14, 22]). Es ist ein Indikator für einen gemischt infiltrativen und obstruktiven Prozess.
3. Die chronische Form der EAA kann sich bei einigen Patienten mit Farmerlunge statt mit einer Fibrose in Form eines zentrilobulären Lungenemphysems äußern.
4. Die fibrotische EAA kann sich selten mit dem klassischen Muster einer „usual interstitial pneumonia“ (UIP) oder nicht spezifischen interstitiellen Pneumonie (NSIP) äußern.
5. Im radiologischen Befund sollte bei der fibrotischen EAA kommentiert werden, ob eine Inflammation vorliegt und wie die Gewichtung zwischen Fibrose und Inflammation ist. Dies hat unmittelbare therapeutische Konsequenz. Bisher hat sich noch keine softwaregestützte/auf künstlicher Intelligenz (KI) basierende Methode etabliert, um diese Gewichtung objektiv vornehmen zu können. Daher muss subjektiv durch die Radiologin/den Radiologen eingeschätzt werden, ob die Inflammation (Milchglastrübung, Milchglasnoduli oder Konsolidierung außerhalber offensichtlicher Fibrose) oder die Zeichen einer Fibrose quantitativ im Vordergrund stehen. Für den behandelnden Pneumologen muss aus dieser Einschätzung ersichtlich sein, ob eher eine antiinflammatorische Therapie, eine antifibrotische Therapie oder eine Kombinationstherapie zielführend ist.

**Tab. 1:** Terminologie der Zeichen in der High-Resolution-Computertomographie (HRCT) bei exogen-allergischer Alveolitis (EAA) und pathophysiologisches Korrelat

HRCT-Zeichen	HRCT-Morphologie	Korrelation
Drei-Dichte-Zeichen (hohe Spezifität)	*Areale mit 3 unterschiedlichen Dichtestufen:* Erhöht: entspricht Milchglas/Konsolidierung Erniedrigt: entspricht Air-Trapping Mittlere Graustufe: entspricht normalem Parenchym	Kombination aus infiltrativem und obstruktivem Prozess sowie nicht betroffenem Lungenparenchym
Honigwaben (nicht dominant)	Gruppierte, zystische Lufträume, die von dicken Wänden umgeben sind	Fibrosierung im Endstadium
Konsolidierung (selten)	Areale vermehrter Dichte ohne erkennbare Anatomie (Gefäße, Bronchialwände)	Inflammation, organisierende Pneumonie
Milchglastrübung	Areale vermehrter Dichte mit noch erkennbarer Anatomie (Gefäße, Bronchialwände)	Inflammation oder feine Fibrose
Milchglasknötchen	Noduli (bis ca. 5 mm Diameter) mit Milchglasdichte, unscharf begrenzt	Inflammatorische Bronchiolitis
Mosaikmuster durch Air-Trapping	Areale mit geografisch konfigurierter abwechselnd hoher und niedriger Dichte. Zunahme der Dichteunterschiede in Exspiration	Obstruktive Bronchiolitis, reflektorische hypoxische Vasokonstriktion
Retikulationen	Irregulär, feinmaschig oder grobmaschig, mit oder ohne Traktionsbronchi(ol)ektasen oder Honigwaben	Fibrosierung vor allem des intralobulären Interstitiums
Traktionsbronchiektasen/-bronchiolektasen	Dilatation der Bronchien/Bronchioli	Irreversible Erweiterung der Atemwege durch den Zug der umgebenden Lungenfibrose
Zysten (in ca. 10–40 % der Fälle)	Zart berandet, bis ca. 2,5 cm Diameter	Folge einer partiellen bronchiolären Obstruktion

**Tab. 2:** High-Resolution-Computertomographie (HRCT)-Morphologie der inflammatorischen exogen-allergischen Alveolitis (EAA)

HRCT-Zeichen	Verteilung
Milchglastrübung (dominant)	Meist diffus, weniger häufig multilokulär
Milchglasknötchen (dominant)	Zentrilobulär
Konsolidierung (selten)	Multilokulär, beidseitig
Air-Trapping	Multilokulär, beidseitig
Drei-Dichte-Zeichen	Multilokulär, beidseitig
Zysten	Innerhalb von Milchglas

**Tab. 3:** High-Resolution-Computertomographie(HRCT)-Morphologie der fibrotischen exogen-allergischen Alveolitis (EAA)

HRCT-Zeichen	Verteilung
Zusätzlich zu inflammatorischen Zeichen oder nur Zeichen der Fibrose	
*Fibrose*	
Retikulation	*Axial*: Meist asymmetrisch – multilokulär, peribronchovaskulär (20 %), peripher *Kraniokaudal*: Oberlappenbetont (10 %), unterlappenbetont (30 %), diffus oder zufällig 60 %, relative Aussparung dorsobasal 20–40 %
Traktionsbronchi(ol)ektasen	
Honigwaben	
*Lungenemphysem* (meist zentrilobulär)	Variabel, Oberlappendominanz möglich

**Abb. 4:**
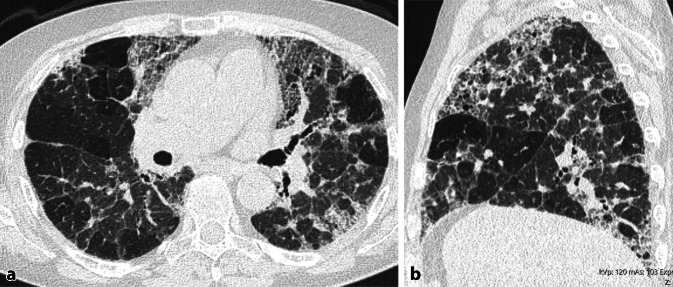
Fibrotische exogen-allergische Alveolitis (EAA) mit viel Inflammation. **a** Axiale und **b** sagittale multiplanare Reformation (MPR) einer nativen High-Resolution-Computertomographie (HRCT). Oberlappenbetonte Fibrose erkenntlich an irreguläre Retikulationen und Traktionsbronchiektasen. Zudem diffus verteiltes Milchglas und Air-Trapping. Beachte auch das Drei-Dichte-Zeichen

**Abb. 5:**
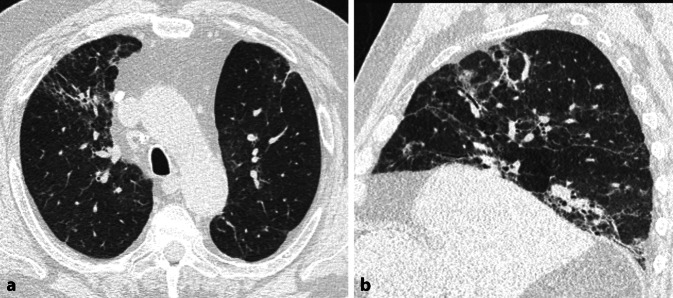
Fibrotische exogen-allergische Alveolitis (EAA) ohne Inflammation. **a** Axiale und **b** sagittale multiplanare Reformation (MPR) einer nativen High-Resolution-Computertomographie (HRCT). Fibrose erkenntlich an irreguläre Retikulationen und Traktionsbronchiektasen. Zudem Air-Trapping. Zeichen einer Inflammation zeigen sich nicht

## Differenzialdiagnose

Radiologisch sind die wichtigsten Differenzialdiagnosen der inflammatorischen EAA:

- die respiratorische Bronchiolitis (jedoch im Gegensatz zur EAA Oberlappendominanz der zentrilobulären Milchglasnoduli und häufig Bronchialwandverdickungen, die keine Begleitzeichen der EAA sind),
- die infektiöse Bronchiolitis (jedoch im Gegensatz zur EAA eher multilokulär als diffuse Verteilung der zentrilobulären Milchglasnoduli, zudem häufig Bronchialwandverdickungen und „tree-in-bud“, die keine Zeichen der EAA sind) und
- die lymphozytäre interstitielle Pneumonie (jedoch im Gegensatz zur EAA wesentlich häufiger und zahlreichere Zysten, kein Air-Trapping und häufig Assoziation mit einer autoimmunologischen Grunderkrankung, vor allem dem Sjögren-Syndrom).

Radiologisch sind die wichtigsten Differenzialdiagnosen der fibrotischen EAA:

- idiopathische Lungenfibrose (IPF) (jedoch im Gegensatz zur EAA subpleural und dorsobasal dominant, Beteiligung der kostophrenischen Winkel, Dominanz von Honigwaben, keine oder nur wenig Zeichen eine Inflammation); das Drei-Dichte-Zeichen hat die höchste Spezifität bei der Differenzierung einer fibrotischen EAA gegenüber einer IPF [4],
- interstitielle Lungenerkrankungen mit dem Muster einer nicht spezifischen interstitiellen Pneumonie (NSIP) (jedoch im Gegensatz zur EAA selten und lediglich diskretes Air-Trapping, nur selten zentrilobuläre Milchglasnoduli, in einem Teil der Fälle relative Aussparung des Subpleuralraums)
- Asbestose (jedoch im Gegensatz zur EAA Expositionsanamnese gegenüber Asbest positiv, Dominanz der Fibrose in den dorsobasalen Unterlappen, asbestbedingte Pleuraveränderungen)

## Prognose

Die Morphologie in der HRCT trägt bei der fibrotischen EAA zur Prognoseabschätzung bei. Mit zunehmendem Ausmaß der Fibrose und mit dem Auftreten von Honigwaben verschlechtert sich die Prognose [1, 11–13]. Wenn Honigwaben nur in 1 oder 2 Lappen auftreten, weist die fibrotische EAA eine bessere Prognose auf als die IPF (medianes Überleben: fibrotische EAA: 8 Jahre gegenüber IPF: 5,2 Jahre; [16]). Wenn Honigwaben in allen 5 Lappen auftreten, gleicht sich das mediane Überleben der beiden Erkrankungen an (2,8 Jahre). Aber auch Retikulationen, Milchglastrübungen und ein Mosaikmuster wurden als unabhängige Prädiktoren für die Mortalität beschrieben [12].

## Fazit für die Praxis

- Im Jahr 2024 ist die erste deutsche Leitlinie zur Diagnostik und Therapie der exogen-allergischen Alveolitis (EAA) erschienen.
- Die Bildgebung nimmt einen hohen Stellenwert bei der Abklärung von PatientInnen mit V. a. EAA ein.
- Die Methode der Wahl ist hierbei die dünnschichtige Volumen-Computertomographie.
- Radiologisch wird eine pragmatische und therapierelevante Einteilung der EAA in eine rein inflammatorische und eine fibrotische EAA vorgeschlagen. Diese Formen sind an der Kombination ausgewählter Zeichen in charakteristischer Verteilung erkennbar.
- Die Wahrscheinlichkeit des Vorliegens einer EAA steigt, je mehr charakteristische Zeichen in typischer Verteilung vorliegen.
- Der radiologische Befund muss Auskunft darüber gegeben, ob eine inflammatorische oder fibrotische EAA vorliegt.
- Im Fall einer fibrotischen EAA muss festgehalten werden, ob eine inflammatorische Komponente vorliegt und wie die Gewichtung zwischen Fibrose und Inflammation ist.
